# The Transcription Factor Lrp of *Pantoea stewartii* subsp. *stewartii* Controls Capsule Production, Motility, and Virulence Important for *in planta* Growth

**DOI:** 10.3389/fmicb.2021.806504

**Published:** 2022-02-14

**Authors:** Holly P. Bartholomew, Guadalupe Reynoso, Brandi J. Thomas, Chase M. Mullins, Chastyn Smith, Irene N. Gentzel, Laura A. Giese, David Mackey, Ann M. Stevens

**Affiliations:** ^1^Department of Biological Sciences, Virginia Tech, Blacksburg, VA, United States; ^2^Department of Horticulture & Crop Science, The Ohio State University, Columbus, OH, United States; ^3^Department of Molecular Genetics and Center for Applied Plant Sciences, The Ohio State University, Columbus, OH, United States; ^4^Center for Emerging, Zoonotic and Arthropod-Borne Pathogens, Virginia Tech, Blacksburg, VA, United States

**Keywords:** corn, maize, *Pantoea stewartii* subsp. *stewartii*, phytopathogen, Stewart’s wilt, transcription factor, xylem

## Abstract

The bacterial phytopathogen *Pantoea stewartii* subsp. *stewartii* causes leaf blight and Stewart’s wilt disease in susceptible corn varieties. A previous RNA-Seq study examined *P. stewartii* gene expression patterns during late-stage infection in the xylem, and a Tn-Seq study using a *P. stewartii* mutant library revealed genes essential for colonization of the xylem. Based on these findings, strains with in-frame chromosomal deletions in the genes encoding seven transcription factors (NsrR, IscR, Nac, Lrp, DSJ_00125, DSJ_03645, and DSJ_18135) and one hypothetical protein (DSJ_21690) were constructed to further evaluate the role of the encoded gene products during *in vitro* and *in planta* growth. Assays for capsule production and motility indicate that Lrp plays a role in regulating these two key physiological outputs *in vitro*. Single infections of each deletion strain into the xylem of corn seedlings determined that Lrp plays a significant role in *P. stewartii* virulence. *In planta* xylem competition assays between co-inoculated deletion and the corresponding complementation or wild-type strains as well as *in vitro* growth curves determined that Lrp controls functions important for *P. stewartii* colonization and growth in corn plants, whereas IscR may have a more generalized impact on growth. Defining the role of essential transcription factors, such as Lrp, during *in planta* growth will enable modeling of key components of the *P. stewartii* regulatory network utilized during growth in corn plants.

## Introduction

*Pantoea stewartii* subsp. *stewartii* is a bacterial phytopathogen that causes leaf blight and Stewart’s wilt disease when it colonizes the apoplast and xylem of corn, respectively. The corn flea beetle, *Chaetocnema pulicaria*, which is endemic to North America, including the mid-Atlantic to midwest regions of the United States (Pataky, 2004), serves as a vector for *P. stewartii*. The bacterium is enteric within the beetle, but it is transferred into the apoplast of the corn leaves during insect feeding. Once inside the plant, the bacteria grow in the apoplast and invade the xylem, where they further proliferate to form a dense biofilm, blocking water transport that leads to wilt during late-stage plant infection. Among the major virulence components of *P. stewartii* are the *hrp*-encoded type III secretion system and the effector WtsE important during both apoplast and xylem infection; a cell wall degrading enzyme (CWDE) thought to be critical for dissemination of *P. stewartii* throughout the plant and in accessing plant nutrients; and the exopolysaccharide (EPS) capsule produced by *P. stewartii* in the xylem that affords protection, enables biofilm formation, and is required for the wilt symptoms in infected plants (Bradshaw-Rouse et al., 1981; Coplin and Cook, 1990; Ham et al., 2006; Mohammadi et al., 2012b; Asselin et al., 2015; Doblas-Ibáñez et al., 2019). Other known virulence factors include capsule pigment, surface motility and adhesins, siderophore production, an RTX toxin, oxidative stress regulation with OxyR and SoxR, outer membrane porins, and Lon protease (Mohammadi et al., 2012a; Burbank et al., 2014; Burbank and Roper, 2014; Kernell Burke et al., 2015; Roper et al., 2015; Duong et al., 2018). Regulation of the bacterium’s transition from the apoplast to the xylem is in large part controlled by a quorum sensing (QS) system, where cell–cell signaling of high cell densities leads to a decrease in motility and an increase in capsule production (Koutsoudis et al., 2006).

In *P. stewartii*, the QS regulon has been a focus of study, including the master transcription factor EsaR plus downstream regulators for capsule production (RcsA) and surface motility (LrhA) (Ramachandran et al., 2014; Kernell Burke et al., 2015; Duong and Stevens, 2017). Interestingly, the *P. stewartii* LrhA regulon is known to be quite different from its homolog in *Escherichia coli* (Kernell Burke et al., 2015). Other published studies in *P. stewartii* reveal regulation of the oxidative stress response signal transduction pathway through the transcription factors OxyR and SoxR (Burbank and Roper, 2014).

To explore the regulation and requirements of *P. stewartii in planta*, an RNA-seq study was used to compare the wild-type transcriptome to that of an *in vitro* culture (Packard et al., 2017). Genes essential for xylem survival *in planta* were subsequently identified using a Tn-Seq approach (Duong et al., 2018). From these data sets, select *P. stewartii* transcription factors were chosen to further study their role in forming a regulatory network *in planta*. Among these were NsrR, IscR, Nac, and Lrp, proteins that are predicted to be involved in regulating nitric oxide stress response, iron–sulfur cluster synthesis, nitrogen assimilation, and the leucine response, respectively, based on their roles in *E. coli* (Muse and Bender, 1998; Schwartz et al., 2001; Brinkman et al., 2003; Bodenmiller and Spiro, 2006; Tucker et al., 2010; Santos et al., 2015). An additional three genes annotated as encoding transcription factors and a hypothetical protein, none of which have been studied in other systems, were also chosen to elucidate their function. It was hypothesized that the products encoded by the selected genes would impact the ability of *P. stewartii* to infect and survive within the corn host xylem environment. The mutant strains and their corresponding complementation or revertant strains were tested through both *in vitro* and *in planta* assays to understand the role of these gene products during the bacterial life cycle *in planta*. Our findings indicate that Lrp is required for the *in planta* lifestyle of *P. stewartii* during growth in the xylem.

## Materials and Methods

### Strains and Growth Conditions

*E. coli* and *P. stewartii* strains were grown at 37 and 30°C, respectively, in LB (10 g/L tryptone, 5 g/L NaCl, 5 g/L yeast extract) broth or 1.5% agar plates. The growth medium was supplemented with the appropriate antibiotics for each strain (see Supplementary Table 1): ampicillin (Amp; 100 μg/mL), chloramphenicol (Cm; 35 μg/mL), gentamycin (Gm; 10 μg/mL), nalidixic acid (Nal; 30 μg/mL), kanamycin (Kan; 50 μg/mL), streptomycin (Str; 100 μg/mL), and tetracycline (Tet; 5 μg/mL). Diaminopimelic acid (DAP) was supplemented in the growth medium for the *E. coli* RHO5 strain (200 μg/mL for broth and 400 μg/mL for agar plates). For *in vitro* growth curves, overnight cultures derived from freezer stocks were subcultured to an optical density at 600 nm (OD_600_) of ∼0.02 in LB supplemented with nalidixic acid, and then growth was monitored in a spectrophotometer over time.

### Gene Selection Criterion

Eight genes were chosen for mutant strain construction, seven annotated as transcription factors and one hypothetical protein. Two annotated transcription factors, *nsrR* and *iscR*, were selected based upon being at least 10-fold reduced in the *in planta* sequencing reads from the Tn-Seq study (Duong et al., 2018) and for the network cross-talk seen between them in *E. coli* (Yeo et al., 2006; Partridge et al., 2009). The genes *lrp* and *nac*, although having missed the 10-fold reduced *in planta* read count in one of the biological replicates from the study, still showed lower reads *in planta* and were chosen based upon their connection to the *nsrR* and *iscR* regulons in *E. coli* (Partridge et al., 2009; Kroner et al., 2019). Each of the three uncharacterized transcription factors as well as the hypothetical protein showed a minimum 10-fold reduction from the Tn-Seq study. Finally, all genes were confirmed to have transcript reads from a previous RNA-Seq study to ensure they were being actively transcribed *in planta* for WT *P. stewartii* (Packard et al., 2017).

### Deletion, Complementation, and Revertant Strain Construction

Two procedures were used for deletion construction, and both of which have been described previously (Kernell Burke et al., 2015; Stice et al., 2020). Briefly, the genes *nsrR*, *iscR*, *nac*, DSJ_03645 (03645), and DSJ_18135 (18135) underwent a markerless deletion construction using the Gateway plasmid transfer system (Life Technologies) with the appropriate primers (Supplementary Table 2), plasmids, and strains (Supplementary Table 1) as previously described (Kernell Burke et al., 2015). The genes *lrp*, DSJ_00125 (00125), and DSJ_21690 (21690) underwent a deletion strategy from methods described by Stice et al. (2020) that was modified as described below. Overlap extension PCR products with *attB* sites for the upstream and downstream regions of the genes of interest were added to the BP Clonase II reaction directly with the suicide vector pR6KT2G. Overnight room temperature BP reactions were transformed into *Eco* MaH1 *pir*^+^ via calcium chloride transformation and LB Gm^10^ plates (1.5% agar) and then colonies patched onto Gm^10^ and Cm^35^ plates. Patches with exclusive growth on Gm^10^ plates were grown overnight in liquid medium, and then plasmid constructs were extracted with a QIAprep spin Miniprep kit (Qiagen), digested via *Xho*I to screen for the expected insertion size, and sent for Sanger sequencing (Fralin Life Sciences Institute; FLSI). Each plasmid was then transformed into the conjugation strain *E. coli* RHO5 on DAP Gm^10^ plates, PCR was used to screen for the expected insert size, and the plasmid was conjugated into DC283 via a 5 μL spot onto LB DAP. After incubating 24 h at 30°C lid-up, each spot was resuspended in 1 mL LB medium, and spread 1× and 10× onto LB Gm^10^ Nal^30^ plates. After 48 h at 30°C, colonies were patched onto LB Gm^10^ Nal^30^ for another 48 h at 30°C. Patches were grown overnight at 30°C in a solution of 75% 1 M sucrose and 25% LB, subcultured to 0.05 OD_600_, and grown until OD_600_ 0.5, plated at 10^–4^–10^–6^ onto LB Nal^30^ and incubated 48 h at 30°C. Colonies were patched onto LB Gm^10^ Nal^30^ and LB Nal^30^ for 24 h at 30°C. Those that grew exclusively on LB Nal^30^ were screened via PCR to determine successful recombination, and amplified fragments of the expected size were sequenced for confirmation.

Complementation strain construction was performed for all deletion strains, except the *lrp* deletion, as described previously (Choi et al., 2005; Kernell Burke et al., 2015). The coding sequence and upstream region to encompass the promoter were inserted into a neutral region downstream of the gene *glmS* in the *P. stewartii* chromosome using the vector system pUC18R6K-mini-Tn7-cat with appropriate primers (Supplementary Table 2).

Revertant strain construction, by which the wild-type *lrp* gene was inserted back into its native locus in the *lrp* deletion strain, was completed through an edited version of the deletion protocol described by Stice et al. (2020) as described above. However, the initial fragment of DNA added to the BP Clonase II reaction directly with the suicide vector pR6KT2G also included the gene of interest along with the areas upstream and downstream of the gene.

### Construction of IG1001

The IG1001 strain of *P. stewartii* was created through homologous recombination of the pING3 plasmid (Supplementary Figure 1) encoding Tet^R^ into WT *P. stewartii* DC283. The pING3 construct was made through a series of steps. First, pING1 was generated by digesting the *Ralstonia* compatibility vector pCOMP-PhII (Monteiro et al., 2012) with *Sal*I and *Nco*I restriction enzymes. The desired 3,799 bp fragment containing the GMI-1 and GMI-2 homologous sequences flanking the tetracycline resistance gene also contained a *Sal*I cut site, so two fragments (1,986 and 1,813 bp, respectively) were ligated into the vector pGEM-T (Promega), also cut with *Sal*I and *Nco*I. Correct orientation of the *Sal*I-*Sal*I fragment insertion was confirmed by selecting for Tet^R^. pING2 was generated by digesting pING1 with *Apa*I and *Nco*I restriction enzymes and ligating in the 1,034 bp Pnss#1 homologous recombination sequence upstream of the GMI-1 sequence. Pnss#1 was amplified through PCR from *P. stewartii* DC283 genomic DNA using primers DMo1110 and DMo1111 (Supplementary Table 2), followed by digestion with *Apa*I and *Nco*I restriction enzymes. pING3 was finally generated by digesting pING2 with *Nde*I and *Sac*I restriction enzymes followed by ligation with the 1,057 bp Pnss#2 homologous recombination sequence (inserted downstream of the GMI-2 sequence). Pnss#2 was amplified through PCR from *P. stewartii* DC283 genomic DNA using primers DMo1113 and DMo1132 (Supplementary Table 2), followed by digestion with *Nde*I and *Sac*I restriction enzymes.

### Capsule Production Phenotypic Assay

The ability of the strains to produce a capsule was tested in duplicate or more as previously described (Kernell Burke et al., 2015). Briefly, strains were grown overnight in the appropriate medium, subcultured to an OD_600_ of 0.05, and grown until they reached an OD_600_ of 0.2. A 2-cm cross streak of each strain was grown on CPG agar plates (0.1% casamino acids, 1% peptone, 1% glucose, 1.5% agar) and incubated at 30°C. After 48 h of growth, 72 h for *lrp*, strains were assessed qualitatively for capsule production levels and photographed with a Bio-Rad Gel Doc imager.

### Surface Motility Phenotypic Assay

Each strain was tested in duplicate or more for surface motility capabilities as described by Kernell Burke et al. (2015). Strains were grown overnight and used to inoculate fresh medium at an OD_600_ of 0.05. Each strain was grown to an OD_600_ of 0.5, and then 5 μL of each strain was spotted onto LB quad-plates with 0.4% agar and supplemented with 0.4% glucose. Plates were incubated for 30 min at room temperature, then stored lid-up in a container with a lid during incubation at 30°C. Plates were photographed with a Bio-Rad Gel Doc imager 48 h, 72 h for *lrp*, after inoculation to qualitatively assess motility capabilities.

### Xylem Virulence Assay

Sweet corn seeds [*Zea mays* cv. Jubilee (2B Seeds, Broomfield, CO) or B73 maize seeds, with the latter used for *lrp* strains] were planted (day 0) in Promix B soil (Premier Tech Horticulture, Rivière-du-Loup, Canada) and grown in a 30°C chamber (Conviron CMP4030) with 16-h light, 8-h dark cycles, ∼200 μE m^–2^s^–1^ light intensity. B73 seeds were produced by self-pollination at the Waterman Farm in Columbus, Ohio. On the fifth day of growth (2–3 leaf stage of the seedlings) plants were inoculated with the *P. stewartii* wild type (WT), deletion strain (Δ), corresponding complementation (Δ/+) or revertant strain or phosphate-buffered saline (PBS; 137 mM NaCl, 2.7 mM KCl, 10 mM Na_2_HPO_4_, and 2 mM KH_2_PO_4_, pH 7.4). As previously described (Kernell Burke et al., 2015), bacterial strains were grown overnight at 30°C and then subcultured to an optical density at 600 nm (OD_600_) of 0.05 and grown until OD_600_ 0.2 when 1 mL of cells were washed with an equal volume of PBS twice at 2 min 10,000 rpm (rotor 5424R) before resuspension in 1 mL PBS. Fifteen plants per inoculum were surface disinfected on the stem at the site of inoculation ∼1 cm above the soil line with 70% ethanol (EtOH) and scratched with a syringe needle on the stem (∼1 cm wound), and 5 μL of washed cells were inoculated into the scratch. Virulence was measured on a 0–5 scale after 10 days postinoculation with 0 = no symptoms, 1 = water-soaking lesions on one leaf, 2 = lesions on two or more leaves, 3 = wilting of one leaf, 4 = wilting of multiple leaves, and 5 = death of the seedling with no symptom-free leaves (Supplementary Figure 2). Student’s *t*-test (*p* ≤ 0.01) was performed in Microsoft Excel software, comparing the disease scores of the WT inoculated plants to the other strains to determine statistical significance.

### Competition Assay

A modified protocol for a previously described competition assay was performed to find relative competition indices (RCI) via counting patched colonies instead of the previously used spread plate colony counting (Duong et al., 2018). Plants were grown and bacterial cultures processed after growth to an OD_600_ of 0.2 on day 5 as described in the virulence assay protocol. After resuspension of the washed cells in 1 mL of PBS, the deletion strain (Nal^R^) was mixed with the corresponding complementation strain (Nal^R^Cm^R^), for all genes except those corresponding to *lrp* in a 1:1 ratio (0.5 mL each strain) and used as the inoculum for six plants. For *lrp*, the strain IG1001 (Nal^R^Tet^R^) was inoculated with the *lrp* deletion because the revertant strain carried the same antibiotic marker as the deletion strain. The initial plant inoculum culture was serially diluted and spread plated on LB Nal^30^, and 100 colonies were replica patched onto Nal^30^ Cm^35^ (or Nal^30^Tet^5^ for IG001) and then LB Nal^30^ to enumerate the complementation (or WT) and deletion strains, respectively. This established the precise input ratio between the two strains at the start of the experiment. Five days after inoculation, the stems were surface disinfected with 70% EtOH and sliced with a sterilized razor blade at the base of the stem and where the leaves emerge. The harvested stem was sliced into ∼2 mm segments and shaken on a rotary platform at 30°C for 1 h in 10 mL of PBS. Each sample was serially diluted and spread on LB Nal^30^ agar plates and 100 colonies patched from these onto Nal^30^ Cm^35^ (or Nal^30^Tet^5^ for IG001) then LB Nal^30^ to enumerate the ratio of complementation or WT and deletion strains that survived *in planta*. This established the precise output ratio between the two strains at the end of the experiment. Both the initial inoculum and final harvested bacterial ratios were used to calculate the RCI as follows: RCI = [deletion patches/complementation patches_output_]/[deletion patches/complementation patches_input_]. A Wilcox pairwise statistical comparison was done via R programming. The RCI values for the experiment performed with the DSJ_21690 strain with an average value closest to one were used as the baseline for comparison to the other strains.

## Results

### Lrp Is Involved in Both Capsule Production and Surface Motility of *Pantoea stewartii*

**FIGURE 1:**
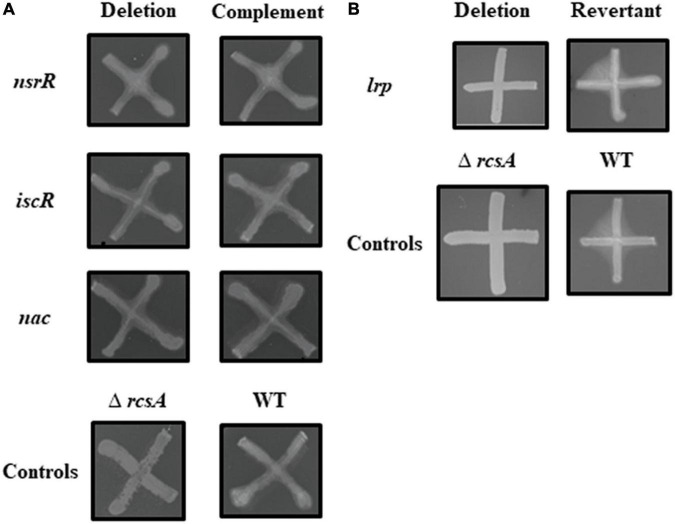
Capsule production by *P. stewartii nsrR*, *iscR*, *nac*, and *lrp* mutant and complementation or revertant strains. All photographs, representative of duplicate or more samples, were taken at the same magnification after incubation in 30°C on CPG agar for 48 h **(A)** or 72 h **(B)** for the indicated strains.

Capsule production was qualitatively examined *in vitro* for each of the *P. stewartii* deletion and complement or revertant strains in comparison to the capsule-producing WT control strain and a Δ*rcsA* control strain, which is known to have an obvious reduction in capsule-producing capabilities (Duong and Stevens, 2017). The Δ*nsrR*, Δ*iscR*, and Δ*nac* strains as well as the four unnamed gene deletion strains produced capsule similar to the WT strain (Figure 1 and Supplementary Figure 3). However, the Δ*lrp* strain, like the Δ*rcsA* strain, was reduced in capsule production even through 72 h, and the *lrp* revertant strain was able to produce WT levels of capsule (Figure 1).

**FIGURE 2:**
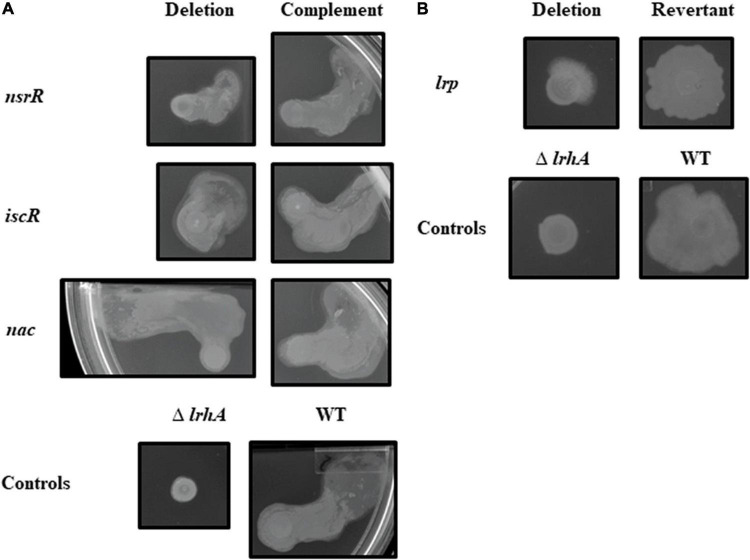
Surface motility of *P. stewartii nsrR*, *iscR*, *nac*, and *lrp* mutant and complementation or revertant strains. All photographs, representative of duplicate or more samples, were taken at the same magnification after incubation in 30°C on LB medium (0.4% agar, 0.4% glucose) for 48 h **(A)** or 72 h **(B)** for the indicated strains.

For the motility assays, the WT strain behaved variably as previously described with either a unidirectional outward expansion or a symmetrical expansion from the point of inoculation (Herrera et al., 2008; Kernell Burke et al., 2015). Thus, a qualitative analysis of motility was performed. The plates were also inoculated with a Δ*lrhA* control strain, which clearly displays reduced motility (Kernell Burke et al., 2015). The Δ*lrp* strain, like Δ*lrhA*, displayed reduced surface motility relative to the WT strain even through 72 h (Figure 2). Besides the Δ*lrp* strain, all other mutant strains tested had motility phenotypes that did not qualitatively differ from the WT strain (Figure 2 and Supplementary Figure 4).

### The Transcription Factor Lrp Impacts *Pantoea stewartii* Xylem Disease Severity

**FIGURE 3:**
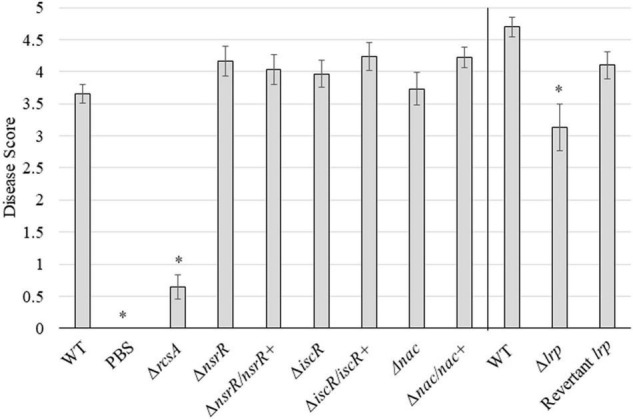
Virulence of *P. stewartii nsrR*, *iscR*, *lrp*, and *nac* mutant and complementation or revertant strains. Average disease score for the indicated *P. stewartii* strains with WT, Δ*rcsA*, and PBS controls. Scores were collected 10 days postinoculation from a minimum of 15 plants (“Jubilee” or “B73” seeds were used for the data shown on left or right side of the vertical bar, respectively). An asterisk (*) represents a significant difference from the appropriate WT strain (*p* ≤ 0.01) using Student’s *t*-test. Error bars were calculated using the standard error for each set.

The WT strain of *P. stewartii* was compared with each deletion and corresponding complement or revertant strain for the ability to cause disease following inoculation into the xylem (Figure 3, Supplementary Figure 5, and Supplementary Table 3). Ten days postinoculation, plants were scored from 0 to 5 on symptoms with the WT strain producing an average score of 3.7 or 4.7 with Jubilee or B73 seeds, respectively. The negative control, PBS, showed an average virulence score of 0, and the Δ*rcsA* control with an anticipated reduction in virulence (Kernell Burke et al., 2015) had an average score of 0.64. The Δ*nsrR*, Δ*iscR*, and Δ*nac* strains were not statistically different than the corresponding WT strain with average virulence scores of 4.2, 4.0, and 3.7, respectively. The Δ*lrp* strain showed a partial reduction in average disease score at 3.1 that was statistically significantly lower compared to the corresponding WT strain (*p* ≤ 0.01). All complement or revertant strains had no statistical difference to the WT levels of disease symptoms. Deletion strains of the three transcription factors of unknown function and the hypothetical protein did not significantly affect *P. stewartii* virulence (Supplementary Figure 5).

### The Transcription Factors Lrp and IscR Play a Role in *Pantoea stewartii* Colonization of Corn

**FIGURE 4:**
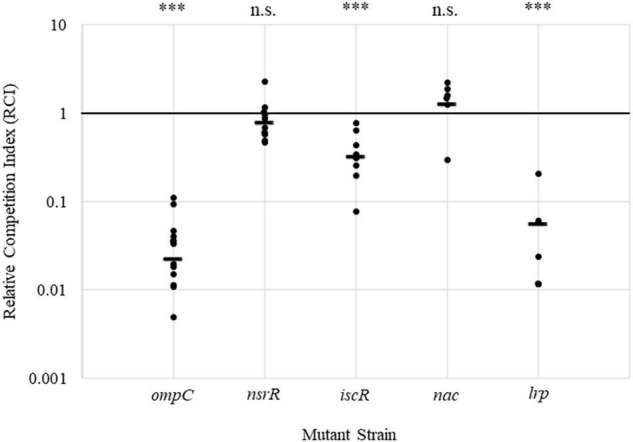
Competition assay for *P. stewartii* mutant strains lacking select transcription regulators. Deletion (Nal^R^) and complementation (Nal^R^/Cm^R^) or WT (Nal^R^/Tet^R^) sets of *nsrR*, *iscR*, *lrp*, and *nac* mutants and the *ompC* control were co-inoculated into the corn seedlings at a 1:1 ratio. The RCI for each set was calculated as the ratio of deletion to complementation or marked WT strains extracted 5 days postinoculation over the ratio of deletion to complementation strains in the inoculum. *N* ≥ 6 per inoculum with the bar representing the average value. A Wilcox pairwise statistical comparison was done via R programming. The RCI values for the experiment performed with the DSJ_21690 strain (see Supplementary Figure 6) with an average value closest to one were used as the baseline for comparison to the other strains. n.s. indicates not significant (*p* > 0.05), and ^***^ indicates significant differences (*p* < 0.005).

To assess the ability of each gene deletion strain to colonize the plant and to validate the original Tn-Seq study findings (Duong et al., 2018), competition assays were performed. Each deletion mutant strain (Nal^R^) was co-inoculated in a 1:1 ratio with the corresponding complementation strain (Nal^R^/Cm^R^) or the WT carrying a chromosomal insertion with a selectable marker (Nal^R^/Tet^R^). The RCI was calculated for each pairing (Figure 4 and Supplementary Figure 6). A competition assay with the *P. stewartii ompC* deletion and complementation strains was used as a positive control based on the colonization requirement of that gene *in planta* (Duong et al., 2018), and results show an average RCI of 0.02, confirming previous work. The *lrp* strain set closely resembled growth observed for *ompC* with an average RCI of approximately 0.05. The *iscR* mutant set had an RCI of 0.3, showing a partial reduction in colonization capabilities of the deletion strain (Figure 4). For the *nsrR* and *nac* strain sets, each had an RCI close to 1 (Figure 4), indicating no defect in colonization capabilities with these mutants. Similarly, DSJ_00125, DSJ_03645, DSJ_18135, and the hypothetical DSJ_21690 had RCI values close to 1, also indicating no obvious colonization defect (Supplementary Figure 6). A Wilcox pairwise comparison of the competition assay data, using the RCI values for DJS_21690 with an average value closest to 1 for the baseline, demonstrated that the RCI values for *ompC*, *iscR*, and *lrp* deletion strain sets were all significantly lower. Overall, the data indicates that Lrp and, to a lesser degree, IscR each contribute to the ability of *P. stewartii* to grow within the xylem *in planta*. Interestingly, growth curves with WT strains DC283 and IG1001, the *iscR* deletion and complement strains, and the *lrp* deletion and revertant strains demonstrated virtually identical growth rates *in vitro* across all strains with the exception of the *iscR* deletion, which trended lower at numerous time points (Supplementary Figure 7). Thus, for *iscR*, growth impacts were seen both *in vitro* and *in planta.* However, for *lrp*, the differences observed *in planta* are likely due to the xylem growth environment and/or the presence of a competing organism.

## Discussion

A previous Tn-Seq study identified 486 genes that were essential for *in planta* xylem survival of *P. stewartia*, and 27 of these genes were annotated transcription factors (Duong et al., 2018). In this study, genes encoding four annotated transcription factors, three putative transcription factors, and one hypothetical protein were further investigated based on the Tn-Seq results and in conjunction with their demonstrated transcription *in planta* from a previous RNA-Seq study (Packard et al., 2017). A combination of *in planta* and *in vitro* techniques were used in an effort to reveal additional components of the regulatory network used by *P. stewartii in planta*.

Competition assays were performed to confirm the initial findings about the essential nature of genes *in planta* from the Tn-Seq study. Surprisingly, six in-frame deletion mutant strains exhibited no competitive disadvantage. Because the 1:1 ratio with two monocultures tested here differs dramatically from the ∼1:40,000 ratio of each mutant within the total Tn-Seq library pool, these differences in community structure may underlie the differences in competitive growth and survival capabilities of the mutants. Polar effects from the transposon insertions, small RNA gene disruption in these regions, temporal gene regulation differences between Tn-Seq and this study, or redundancy in the bacterial networks could have all contributed to the differences observed between the two studies regarding competitive advantages. However, it was demonstrated that deletions in *lrp* and, to a lesser degree *iscR*, decreased the growth rate of *P. stewartii in planta*. A growth defect was also observed *in vitro* for *iscR*, suggesting it might play a generalized role in growth whereas *lrp* appears to specifically impact *in planta* growth. Further, Lrp controls two key outputs, motility and capsule production, necessary for the full virulence of *P. stewartii.*

IscR is a transcription repressor involved in iron metabolism and oxidative stress response in many bacteria, including *E. coli* (Runyen-Janecky et al., 2008). In *E. coli*, IscR functions both without an iron sulfur cluster (apo-IscR) or with the addition of a [2Fe-2S] cluster (holo-IscR). Both forms of the protein are required under normal cellular conditions to balance the Fe-S cluster abundance in the bacterium. Under iron-limited conditions or in cases of oxidative stress, the apo-IscR activates a second Fe-S biosynthesis pathway encoded by the *suf* operon (Mettert and Kiley, 2014; Santos et al., 2015). The gene encoding IscR is required for virulence in several biofilm-producing organisms, including *Klebsiella pneumoniae* and the plant pathogen *Xanthomonas oryzae* (Wu et al., 2014; Fuangthong et al., 2015). Additionally, the type III secretion system (T3SS) in *Yersinia pseudotuberculosis* is known to be influenced by IscR (Miller et al., 2014). In *K. pneumonia*, IscR is demonstrated to be involved in both capsule biosynthesis as well as iron acquisition (Wu et al., 2014). Previous studies in *P. stewartii* show that iron acquisition influences motility of the organism, and loss of siderophore production (i.e., the *iucA* operon) reduces bacterial virulence *in planta* (Burbank et al., 2014). Recent work also reveals a reduction in iron availability within the xylem sap during *in planta* colonization of *P. stewartii*, hypothesized as a plant defense response to the infection (Doblas-Ibáñez et al., 2019). Although a virulence phenotype was not evident in this study for Δ*iscR*, perhaps ties to the iron acquisition system in the reduced iron environment contributed to the reduction in colonization capabilities for Δ*iscR*.

The Lrp transcriptional regulator is a global regulator originally named for its role in the leucine response of *E. coli* (Brinkman et al., 2003). However, recent studies show that Lrp may actually regulate more than a third of all genes within *E. coli* either directly or indirectly under a variety of conditions, including numerous instances of leucine-independent Lrp binding (Shimada et al., 2015; Kroner et al., 2019). Among the identified direct targets are Nac (nitrogen assimilation control), LrhA (motility), SoxS (superoxide stress), and ArgR (arginine biosynthesis), indicating ties to a wide variety of physiological responses within the bacteria involved in stress, navigation, and metabolism. Previously, Lrp has been demonstrated to play a role in the virulence of some plant pathogens, including *Xanthomonas* spp. (Cubero and Graham, 2004), *Acidovorax avenae* (Kondo et al., 2017) and *Erwinia amylovora* (Schachterle and Sundin, 2019). Similarly, our findings indicate critical roles of Lrp for *P. stewartii* with regard to capsule production, motility, growth, and virulence.

The Δ*lrp* mutant revealed a reduction in virulence of *P. stewartii in planta* and in both capsule production and motility *in vitro*. From previous studies in *P. stewartii* (von Bodman et al., 1998; Minogue et al., 2005; Koutsoudis et al., 2006; Ramachandran and Stevens, 2013), capsule and motility regulation are linked through the QS regulator EsaR and the downstream regulators RcsA and LrhA, both of which are important for virulence. Additionally, Lrp in *E. coli* is shown to regulate *lrhA* (Shimada et al., 2015; Kroner et al., 2019). Thus, the role of *lrp* in virulence may be mediated through its connections with capsule and motility. It would be interesting to see how the regulon of the QS system interacts with the regulon of Lrp, as there are numerous predicted links indicating possible cross-talk between these systems, including *lysP*, the lysine permease (Ruiz et al., 2011; Ramachandran et al., 2014). In addition, Lrp in *E. coli* also has connections to oxidative stress via regulation of *soxS*, and SoxRS regulation is also required for virulence in *P. stewartii* (Burbank and Roper, 2014; Kroner et al., 2019). Lrp appears to be a critical master regulator for the *in planta* lifestyle of *P. stewartii*. Future work understanding the *P. stewartii* Lrp regulon will be important to further elucidating its role during *in planta* infection and may provide broader insights applicable to other vascular phytopathogens.

## Data Availability Statement

The original contributions presented in the study are included in the article/Supplementary Material, further inquiries can be directed to the corresponding author.

## Author Contributions

HB, GR, IG, LG, DM, and AS conceived and designed the experiments. HB, GR, BT, CM, CS, IG, LG, and AS performed the experiments. HB, GR, DM, and AS analyzed the data and drafted the manuscript. HB and GR prepared the figures and tables. All authors reviewed and approved the manuscript.

## Conflict of Interest

The authors declare that the research was conducted in the absence of any commercial or financial relationships that could be construed as a potential conflict of interest.

## Publisher’s Note

All claims expressed in this article are solely those of the authors and do not necessarily represent those of their affiliated organizations, or those of the publisher, the editors and the reviewers. Any product that may be evaluated in this article, or claim that may be made by its manufacturer, is not guaranteed or endorsed by the publisher.
