# Supplementary material for: The Transcription Factor Lrp of Pantoea stewartii subsp. stewartii Controls Capsule Production, Motility, and Virulence Important for in planta Growth
Source: Front Microbiol. 2022 Feb 14;12:806504. doi: 10.3389/fmicb.2021.806504 (PMC8882988; doi:10.3389/fmicb.2021.806504)
Supplement: Supplementary file 1 [file Data_Sheet_1.docx]

**Supplementary Tables and Figures**

**Table S1. Strains used in this study**

| **Strains** | **Genotype and notes^a^** | **References** |
| --- | --- | --- |
| ***Pantoea stewartii* strains** | | |
| DC283 | Wild-type strain; Nal^R^ | Dolph et al., 1988 |
| IG1001 | DC283-derived recipient strain (homologous recombination with pING3); Nal^R^ Tet^R^ | This study |
| ∆*rcsA* | Unmarked deletion of *rcsA* coding sequence from DC283; Nal^R^ | Duong & Stevens, 2017 |
| ∆*lrhA* | Unmarked deletion of *lrhA* coding sequence from DC283; Nal^R^ | Kernell Burke et al., 2015 |
| ∆*ompC* | Unmarked deletion of both *ompC* coding sequence from DC283; Nal^R^ | Duong, Jensen, & Stevens, 2018 |
| ∆*ompC/ompC^+^* ^­^ | ∆*ompC* with chromosomal complementation of *ompC* and its promoter downstream of *glmS*; Nal^R^ Cm^R^ | Duong, Jensen, & Stevens, 2018 |
| ∆*nsrR* | Unmarked deletion of *nsrR* coding sequence from DC283; Nal^r^ | This study |
| ∆*nsrR/nsrR^+^* | *∆nsrR* with chromosomal complementation of *nsrR* and its promoter downstream of *glmS*; Nal^R^ Cm^R^ | This study |
| *∆iscR* | Unmarked deletion of *iscR* coding sequence from DC283; Nal^R^ | This study |
| ∆*iscR/iscR^+^* | *∆iscR* with chromosomal complementation of *iscR* and its promoter downstream of *glmS*; Nal^R^ Cm^R^ | This study |
| *∆lrp* | Unmarked deletion of *lrp* coding sequence from DC283; Nal^R^ | This study |
| Revertant *lrp* | Unmarked reversion of *lrp* by homologous recombination in *∆lrp* | This study |
| ∆*nac* | Unmarked deletion of *nac* coding sequence from DC283; Nal^R^ | This study |
| ∆*nac/nac^+^* | *∆nac* with chromosomal complementation of *nac* and its promoter downstream of *glmS*; Nal^R^ Cm^R^ | This study |
| ∆DSJ_00125 | Unmarked deletion of DSJ_00125 coding sequence from DC283; Nal^R^ | This study |
| ∆DSJ_00125/DSJ_00125*^+^* | ∆DSJ_00125/DSJ_00125*^+^* with chromosomal complementation of DSJ_00125 and its promoter downstream of *glmS*; Nal^R^ Cm^R^ | This study |
| ∆DSJ_03645 | Unmarked deletion of DSJ_03645 coding sequence from DC283; Nal^R^ | This study |
| ∆DSJ_03645/DSJ_03645*^+^* | ∆DSJ_03645/DSJ_03645*^+^* with chromosomal complementation of DSJ_03645 and its promoter downstream of *glmS*; Nal^R^ Cm^R^ | This study |
| ∆DSJ_18135 | Unmarked deletion of DSJ_18135 coding sequence from DC283; Nal^R^ | This study |
| ∆DSJ_18135/DSJ_18135*^+^* | ∆DSJ_18135/DSJ_18135*^+^* with chromosomal complementation of DSJ_18135 and its promoter downstream of *glmS*; Nal^R^ Cm^R^ | This study |
| ∆DSJ_21690 | Unmarked deletion of DSJ_21690 coding sequence from DC283; Nal^R^ | This study |
| ∆DSJ_21690/DSJ_21690*^+^* | ∆DSJ_21690/DSJ_21690*^+^* with chromosomal complementation of DSJ_21690 and its promoter downstream of *glmS*; Nal^R^ Cm^R^ | This study |
| ***Escherichia coli* strains** | | |
| DH5α *λpir* | *F^-^* *endA1 glnV44 thi-1 recA1 relA1 gyrA96 deoR nupG Φ80dlacZΔM15 Δ(lacZYA-argF)U169 hsdR17(rK- mK+) λpir* | Kvitko et al., 2012 |
| MaH1 | *attTn7 pir116* R6K replicon plasmids, DH5α derivative | Kvitko et al., 2012 |
| RHO5 | *F^-^, λpir+, thi-1, thr-1, leuB6, lacY1, tonA21, glnV44, recA, yfcU::Mu, Δasd::FRT, glvB::RP4-2-TcR::Mu, ΔaphA::FRT (Km^S^), attTn7::pir116*  DAP-dependent conjugation strain | Kvitko et al., 2012 |
| S17-1 *λpir* | *recA pro hsdR RP4-2-Tc::Mu-Km::Tn7 λpir* | Labes et al., 1990 |
| Top 10 | *F^-^* *mcrA* Δ(*mrr-hsdRMS-mcrBC*) *Φ80dlacZΔM15* Δ*lacX*74 *deoR recAI araD139* Δ(*ara-leu)7697 galU galK rpsL (*Str^r^*) endA1 nupG* | Grant et al., 1990 |
| **Plasmids** | | |
| pAUC40 | Suicide vector pKNG101::*attR*-*ccdB*-Cm^R^; Cm^R^, Str^R^, *sacB* | Carlier et al., 2009 |
| pCOMP-PhII | *Ralstonia* compatibility vector specific for *Ralstonia solanacearum* UW551 phytotype | Monteiro et al., 2012 |
| pDONR201 | Entry vector in the Gateway system, Kn^R^ | Life Technologies |
| pEVS104 | Conjugative helper plasmid, *tra trb;* Kn^R^ | Stabb & Ruby, 2002 |
| pGEM-T | Cloning vector, Amp^R^ | Promega |
| pING1 | pGEM-T with SalI/NcoI fragment cut from pCOMP-PhII; Amp^R^ | This study |
| pING2 | pING1 with *P. stewartii* #1 homologous sequence inserted with ApaI and NcoI | This study |
| pING3 | pING2 with *P. stewartii* #2 homologous sequence inserted with NdeI and SacI | This study |
| pR6KT2G | Gateway-derivative of pR6KT2, a Tn7 vector for chromosomal integration into the intergenic region downstream of *glmS*; sacB, gus, Cm^R^, Gm^R^ | Stice et al., 2020 |
| pUC18R6K-mini-Tn7-cat | Tn7 vector for chromosomal integration into the intergenic region downstream of *glmS*; Cm^R^, Amp^R^ | Choi et al., 2005 |

**^a^** Amp^R^, ampicillin resistance; Cm^R^, chloramphenicol resistance; Gm^R^, gentamycin resistance Kn^R^, kanamacyin resistance; Nal^R^, nalidixic acid resistance; Tet^R^, tetracycline resistance; Str^R^, streptomycin resistance; DAP, diaminopimelic acid

**Table S2. Primers used for this study^a^**

| **Deletion construction** | | |
| --- | --- | --- |
| NAC-UPF | GTCGACAATACGTGTACGCCATCG | Amplify 1 kb region upstream of *nac* |
| NAC-UPR | AGTGGAATATAGGCGGCCGCTAAGTTCATCTTGCCTCCG |  |
| NAC-DNF | GCGGCCGCCTATATTCCACTTTCTGTTTATCCCTCTCAAGC | Amplify 1 kb region downstream of *nac* |
| NAC-DNR | GGATCCCTGATCGGCCAGAATACC |  |
| NAC-1kbUPF-attB1 | GGGGACAAGTTTGTACAAAAAAGCAGGCTGTCGACAATACGTGTACGCCATCG | Amplify 2 kb deletion fragment of *nac* with flanking *attB* sites |
| NAC-1kbDNR-attB2 | GGGGACCACTTTGTACAAGAAAGCTGGGTGGATCCCTGATCGGCCAGAATACC |  |
| UP- NAC-F | GCCTCATCCTCGGTGAAGATTGC | Screen/sequence mutants for *nac* deletion |
| IN- NAC-F | TGATGAATGCCGGTCAGGTGG |  |
| DN- NAC-R | GGACGCACAGAGAGGACAGC |  |
| NSRR-UPF | GTCGACCGGTACCTATCCCTATGTG | Amplify 1 kb region upstream of *nsrR* |
| NSRR-UPR | AGTGGAATATAGGCGGCCGCCCTCTGTAATACTGGTTAGTCTG |  |
| NSRR-DNF | GCGGCCGCCTATATTCCACTGAAATTGTATTGTCTGAACCGC | Amplify 1 kb region downstream of *nsrR* |
| NSRR-DNR | GGATCCATAGTGACTAACGGCAACTG |  |
| NSRR-1kbUPF-attB1 | GGGGACAAGTTTGTACAAAAAAGCAGGCTGTCGACCGGTACCTATCCCTATGTG | Amplify 2 kb deletion fragment of *nsrR* with flanking *attB* sites |
| NSRR-1kbDNR-attB2 | GGGGACCACTTTGTACAAGAAAGCTGGGTGGATCCATAGTGACTAACGGCAACTG |  |
| UP- NSRR-F | AGCCGATCTATGAAACCCAGCC | Screen/sequence mutants for *nsrR* deletion |
| IN- NSRR-F | GCCTTACATGATGCCGTGCAG |  |
| DN- NSRR-R | AGATCAAGACGTTCAGGCAGGG |  |
| ISCR-UPF | GTCGACAAAGTGCCATTATCATGTGG | Amplify 1 kb region upstream of *iscR* |
| ISCR -UPR | AGTGGAATATAGGCGGCCGCACGTCCTTTGGATGTCAG |  |
| ISCR -DNF | GCGGCCGCCTATATTCCACTGGAAATCAACGTTAACCTCC | Amplify 1 kb region downstream of *iscR* |
| ISCR -DNR | GGATCCATCTCTTCTTCACGCACC |  |
| ISCR -1kbUPF-attB1 | GGGGACAAGTTTGTACAAAAAAGCAGGCTGTCGACAAAGTGCCATTATCATGTGG | Amplify 2 kb deletion fragment of *iscR* with flanking *attB* sites |
| ISCR -1kbDNR-attB2 | GGGGACCACTTTGTACAAGAAAGCTGGGTGGATCCATCTCTTCTTCACGCACC |  |
| UP- ISCR -F | ACGACCTGGAGCGTTTCTACC | Screen/sequence of mutants for *iscR* deletion |
| IN- ISCR -F | GACCTGAGCGTCCGTATTAGCG |  |
| DN- ISCR -R | ACAGGCAGTTCAGCGAGATCG |  |
| LRP-UPF-attB1 | GGGGACAAGTTTGTACAAAAAAGCAGGCTGTCGACATGCATACGTTCCATCAGC | Amplify 1 kb region upstream of *lrp* with *attB* site |
| LRP-UPR | TAACTCGAGTGCCTAGGTATGGTACCTCCTGTCGAGATCCTTACC |  |
| LRP-DNF | AGGTACCATACCTAGGCACTCGAGTTAAGCAATCGTCTGGTGATC | Amplify 1 kb region downstream of *lrp* with *attB* site |
| LRP-DNR-attB2 | GGGGACCACTTTGTACAAGAAAGCTGGGTGGATCCAAGGTTAACCATGACCAGC |  |
| UP- LRP-F | ACGGAAGGGACGGTTCTGC | Screen/sequence of mutants for *lrp* deletion |
| IN- LRP-F | CGTGCCTGATATGTCCGCTTACC |  |
| DN- LRP-R | ATGCCAGGGTTCATCGTGACG |  |
| DSJ_00125-UPF-attB1 | GGGGACAAGTTTGTACAAAAAAGCAGGCTGTCGACAAGATATTACTGGCGCTGG | Amplify 1 kb region upstream of DSJ_00125 with *attB* site |
| DSJ_00125-UPR | TAACTCGAGTGCCTAGGTATGGTACCTCTAATAGTGGCGCGAGAAG |  |
| DSJ_00125-DNF | AGGTACCATACCTAGGCACTCGAGTTATATTGAAGGCCACGATCATC | Amplify 1 kb region downstream of DSJ_00125 with *attB* site |
| DSJ_00125-DNR-attB2 | GGGGACCACTTTGTACAAGAAAGCTGGGTGGATCCATATTCCGCAATCAGCATG |  |
| UP- DSJ_00125-F | GTCGTCTACCCGATGAGCGC | Screen/sequence of mutants for DSJ_00125 deletion |
| IN- DSJ_00125-F | GGGTTTGATGAGCTGGAGTGGG |  |
| DN- DSJ_00125-R | ACCGCTGCTTTGACGTGCAC |  |
| DSJ_03645-UPF | GTCGACCGATCATTTCATCATCTTCAACC | Amplify 1 kb region upstream of DSJ_03645 |
| DSJ_03645-UPR | AGTGGAATATAGGCGGCCGCTTAGTTCAACCACGCGTG |  |
| DSJ_03645-DNF | GCGGCCGCCTATATTCCACTAATCTGCTCTGTGATGTGC | Amplify 1 kb region downstream of DSJ_03645 |
| DSJ_03645-DNR | GGATCCAAGGCCACAAAGATGATCG |  |
| DSJ_03645-1kbUPF-attB1 | GGGGACAAGTTTGTACAAAAAAGCAGGCTGTCGACCGATCATTTCATCATCTTCAACC | Amplify 2 kb deletion fragment of DSJ_03645 with flanking *attB* sites |
| DSJ_03645-1kbDNR-attB2 | GGGGACCACTTTGTACAAGAAAGCTGGGTGGATCCAAGGCCACAAAGATGATCG |  |
| UP- DSJ_03645-F | TAGCGCGCTAAAGTGTGTTCCTGC | Screen/sequence mutants for DSJ_03645 deletion |
| IN- DSJ_03645-F | CCTATTTGCCTTCGTACATGGTGCAGG |  |
| DN- DSJ_03645-R | TTCAGAGTCAGGTGCTTCCGACG |  |
| DSJ_18135-UPF | GTCGACCCAGAATGATTAACACCATCCC | Amplify 1 kb region upstream of DSJ_18135 |
| DSJ_18135-UPR | AGTGGAATATAGGCGGCCGCGCTGGATGATTGTTGAGTCAT |  |
| DSJ_18135-DNF | GCGGCCGCCTATATTCCACTGGCTCTGATTCGTGAAATTGG | Amplify 1 kb region downstream of DSJ_18135 |
| DSJ_18135-DNR | GGATCCCGTCTGCATTTCTTCCACC |  |
| DSJ_18135-1kbUPF-attB1 | GGGGACAAGTTTGTACAAAAAAGCAGGCTGTCGACCCAGAATGATTAACACCATCCC | Amplify 2 kb deletion fragment of DSJ_18135 with flanking *attB* sites |
| DSJ_18135-1kbDNR-attB2 | GGGGACCACTTTGTACAAGAAAGCTGGGTGGATCCCGTCTGCATTTCTTCCAC |  |
| UP- DSJ_18135-F | GCCGAAACAATCCAGCTTCCG | Screen/sequence mutants for DSJ_18135 deletion |
| IN- DSJ_18135-F | CGCTCGATACGCTGTTTGGC |  |
| DN- DSJ_18135-R | CACGCGCCTTAATGCCGG |  |
| DSJ_21690-1kbUPF-attB1 | GGGGACAAGTTTGTACAAAAAAGCAGGCTGTCGACAAATTCAGCTCTCAGGGC | Amplify 1 kb region upstream of DSJ_21690 with *attB* site |
| DSJ_21690-UPR | AGTGGAATATAGGCGGCCGCGGGTAGAGAGAATGCTTAAAGC |  |
| DSJ_21690-DNF | GCGGCCGCCTATATTCCACTTACAACGATGGCAACTATCAC | Amplify 1 kb region downstream of DSJ_21690 with *attB* site |
| DSJ_21690-1kbDNR-attB2 | GGGGACCACTTTGTACAAGAAAGCTGGGTGGATCCACTCTGTCATTAAATACGTCACC |  |
| UP- DSJ_21690-F | ACTGTTTACCTGCGGAACTGGG | Screen/sequence of mutants for DSJ_21690 deletion |
| IN- DSJ_21690-F | TTAGTGGTGTCGCCGGTGC |  |
| DN- DSJ_21690-R | CCTGCGCCTGTTGATTTGTTACCG |  |
| **Chromosomal complementation construction** | | |
| C_NAC_F2_SacI | GAGCTCAACAGGGTAGTAAACGTTTCGG | Amplify promoter and coding region of *nac,* and screen conjugants |
| C_NAC_R1_XhoI | CTCGAGCTATAAACAGAACGCTGTGTTTAGC |  |
| C_NSRR_F_SacI | GAGCTCACCCATTCAGCGCGTAATC | Amplify promoter and coding region of *nsrR,* and screen conjugants |
| C_NSRR_R1_XhoI | CTCGAGCTATTTGACATTACGGTTCCTCC |  |
| C_ISCR_F_SacI | GAGCTCAAACGGCCCTGAATAGTTG | Amplify promoter and coding region of *iscR,* and screen conjugants |
| C_ISCR_R_XhoI | CTCGAGCTAGGAGAAGCAGAGGATTAGG |  |
| C_LRP_F_SacI | GAGCTCGCGAATAACTTAGTCTGAAC | Amplify promoter and coding region of *lrp,* and screen conjugants |
| C_LRP_R_XhoI | CTCGAGCTATGGTCCTGTATGTATTCAC |  |
| C_00125_F_SacI | GAGCTCTTATTGCGATTCGTCAGGC | Amplify promoter and coding region of DSJ_00125*,* and screen conjugants |
| C_00125_R_XhoI | CTCGAGCTAGTAAGTGAAGGGAACAATCTG |  |
| C_03645_F_SacI | GAGCTCTTGTTGTTTCCTCTCGCCATGG | Amplify promoter and coding region of DSJ_03645*,* and screen conjugants |
| C_03645_R_XhoI | CTCGAGCTATGTATTGCAACAATCAGCGACC |  |
| C_18135_F_SacI | GAGCTCGGTTCGATGTATTGAGATGCG | Amplify promoter and coding region of DSJ_18135*,* and screen conjugants |
| C_18135_R_XhoI | CTCGAGCTAACTCATCCTTCAACAGCG |  |
| C_21690_F_SacI | GAGCTCAGCCTGATCGACCATAAATTTCC | Amplify promoter and coding region of DSJ_21690*,* and screen conjugants |
| C_21690_R_XhoI | CTCGAGCTATTATTTGTACAACTCAGCGGTGG |  |
| **Construction of IG1001** | | |
| DMo1110 | CGGGCCCCACATCCAGATCATCGCCTGTG | Forward *Pnss*#1 homologous sequence cloning primer-contains ApaI site at 5’ end |
| DMo1111 | AGACACCATGGACTGGAGCAATGCAGTAGCAAG | Reverse *Pnss*#1 homologous sequence cloning primer-contains NcoI site at 5’end |
| DMo1113 | GGAGCTCGAGGTACCTGAGCCATCCGC | Reverse *Pnss*#2 homologous sequence cloning primer-contains SacI site at 5’ end |
| DMo1132 | TACTCATATGGCACGCTGTTGCTCGCTG | Forward *Pnss* #2 homologous sequence cloning primer-contains NdeI stie at 5’ end |

**^a^** All sequences 5’ to 3’

**Table S3. Virulence score distribution for corn plants inoculated with individual *P. stewartii* strains^a^**

| **Strain** | **0-1** | **1-2** | **2-3** | **3-4** | **4-5** |
| --- | --- | --- | --- | --- | --- |
| WT DC283 | 3 | 3 | 11 | 20 | 11 |
| PBS | 31 | 0 | 0 | 0 | 0 |
| ∆*iscR* | 0 | 1 | 1 | 8 | 5 |
| ∆*iscR/iscR*+ | 0 | 1 | 1 | 5 | 8 |
| ∆*nac* | 0 | 1 | 5 | 3 | 6 |
| ∆*nac/nac*+ | 0 | 0 | 1 | 6 | 8 |
| ∆*nsrR* | 0 | 1 | 1 | 6 | 7 |
| ∆*nsrR/nsrR*+ | 0 | 1 | 2 | 5 | 7 |
| ∆DSJ_00125 | 0 | 1 | 1 | 6 | 7 |
| ∆DSJ_00125/DSJ_00125+ | 2 | 1 | 1 | 5 | 6 |
| ∆DSJ_03645 | 0 | 0 | 5 | 3 | 7 |
| ∆DSJ_03645/DSJ_03645+ | 2 | 0 | 1 | 2 | 10 |
| ∆DSJ_18135 | 0 | 2 | 1 | 7 | 5 |
| ∆DSJ_18135/DSJ_18135+ | 1 | 0 | 3 | 3 | 8 |
| ∆DSJ_21690 | 0 | 0 | 2 | 7 | 6 |
| ∆DSJ_21690/DSJ_21690+ | 1 | 0 | 0 | 7 | 7 |
| ∆*rcsA* | 10 | 2 | 1 | 0 | 0 |
| ∆*lrp*^b^ | 3 | 1 | 2 | 6 | 3 |
| Revertant *lrp*^b^ | 0 | 0 | 2 | 7 | 6 |
| WT DC283^b^ | 0 | 0 | 1 | 2 | 12 |

^a^ Scores were collected ten days post-inoculation from a minimum of 15 plants (except ∆*rcsA*, with 13 plants).

^b^ Indicated strains were inoculated into ‘B73’ corn seedlings. (All other strains were inoculated into ‘Jubilee’ corn seedlings.)


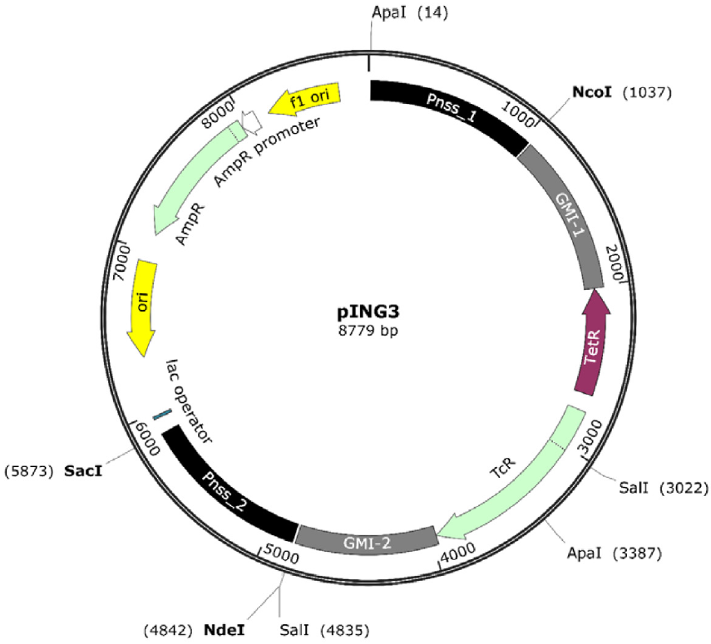


**Figure S1. Vector map of pING3 used for *lrp* revertant strain creation.** See text for details.


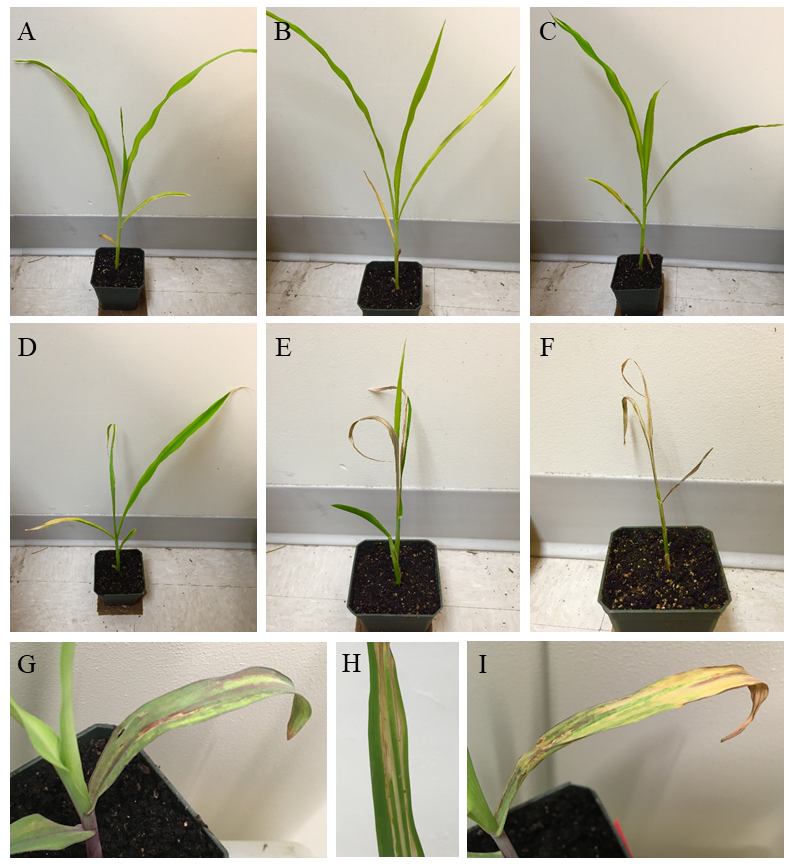


**Figure S2.** **Examples of Stewart’s wilt symptom severity.** Scoring of symptom severity is based on 1-point increments, with (A) 0 = no symptoms, (B) 1 = water-soaked lesions on one leaf, (C) 2 = lesions on two or more leaves, (D) 3 = wilting of one leaf, (E) 4 = wilting of multiple leaves, (F) 5 = death of the seedling with no symptom-free leaves. (G & H) A close up view of the leaf-blight lesions and (I) wilt symptoms.

**
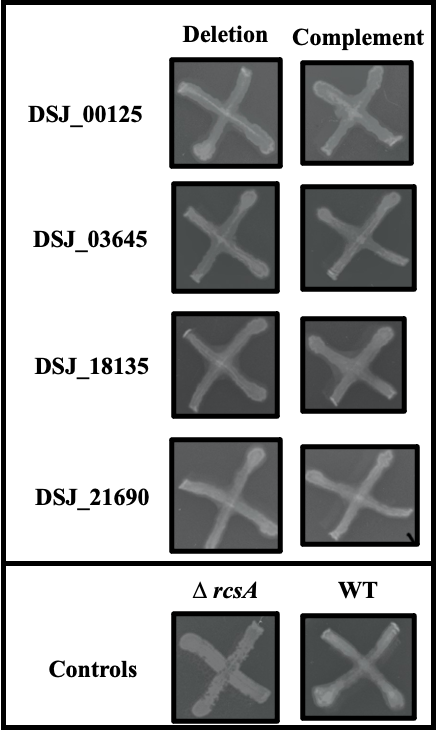
**

**Figure S3. Capsule production phenotype of DSJ_00125, DSJ_03645, DSJ_18135, and DSJ_21690 mutant and complementation strains with controls.** All photographs, representative of duplicate or more samples, were taken at the same magnification after a 48 h incubation at 30°C on CPG agar.


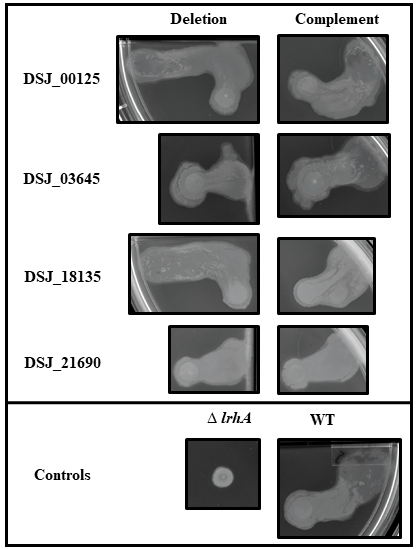


**Figure S4. Surface motility phenotype of DSJ_00125, DSJ_03645, DSJ_18135, and DSJ_21690 strains with controls.** All photographs, representative of duplicate or more samples, were taken at the same magnification after a 48 h incubation at 30°C on LB medium (0.4% agar, 0.4% glucose).


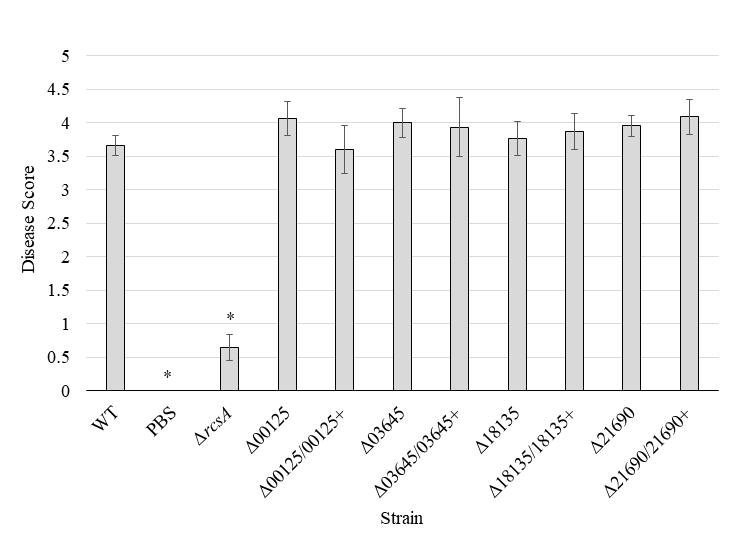


**Figure S5. Virulence of *P. stewartii* DSJ_00125, DSJ_03645, DSJ_18135, and DSJ_21690 mutant and complementation strains.** Average disease score for the indicated *P. stewartii* strains with WT, ∆*rcsA* and PBS controls. Scores were collected 10 days postinoculation from a minimum of 15 plants. An asterisk (*) represents a significant difference from the WT strain (p ≤ 0.01) using Student’s T-Test. Error bars were calculated using the standard error for each set.


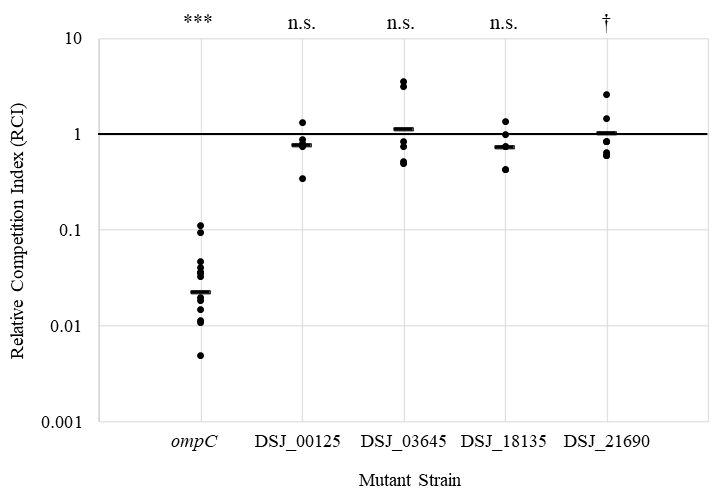


**Figure S6. Competition assay for *P. stewartii* mutant strains lacking select unannotated genes.** Deletion (Nal^R^) and complementation (Nal^R^/Cm^R^) strain sets of DSJ_00125, DSJ_03645, DSJ_18135, and DSJ_21690 mutants, and the *ompC* control, were co-inoculated into the corn seedlings at a 1:1 ratio. The RCI for each set was calculated as the ratio of deletion to complementation strains extracted 5 days postinoculation over the ratio of deletion to complementation strains in the inoculum. N ≥ 5 per inoculum with the bar representing the average value. A Wilcox pairwise statistical comparison was done via R programming. The RCI values for the experiment performed with the DSJ_21690 strain (†) with an average value closest to one were used as the baseline for comparison to the other strains. n.s. indicates not significant (p>0.05), and *** indicates significant differences (p<0.005).


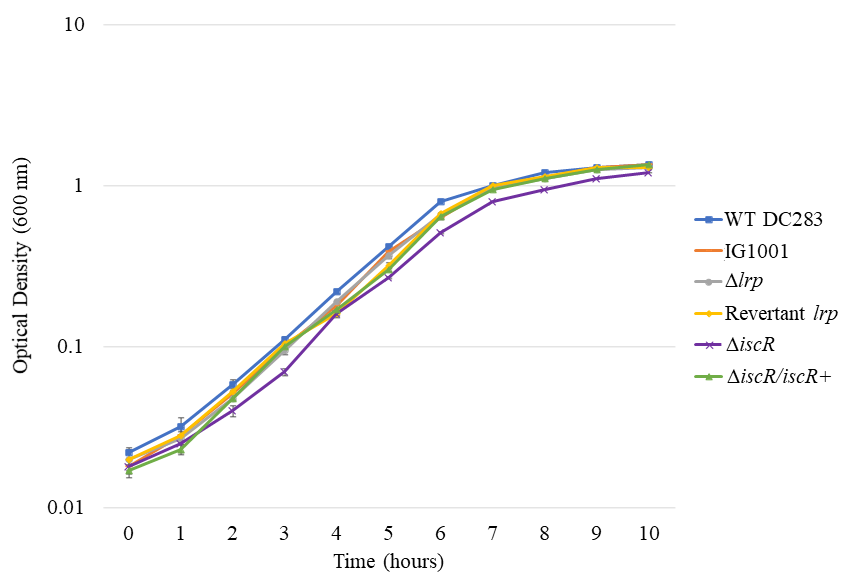


**Figure S7. Growth curve of *P. stewartii* monoculture strains, as indicated.** Growth in LB medium was monitored every hour over 10 hours using optical density at 600 nm. Error bars represent standard error across triplicate samples.

**Supplementary References**

Carlier A, Burbank L, von Bodman SB. 2009. Identification and characterization of three novel EsaI/EsaR quorum-sensing controlled stewartan exopolysaccharide biosynthetic genes in *Pantoea stewartii* ssp. *stewartii*. *Molecular Microbiology* 74:903–913. doi: 10.1111/j.1365-2958.2009.06906.x

Choi KH, Gaynor JB, White KG, Lopez C, Bosio CM, Karkhoff-Schweizer RR, Schweizer HP. 2005. A Tn7-based broad-range bacterial cloning and expression system. Nature Methods 2:443-448.

Dolph, PJ, Majerczak, DR, and Coplin, DL. 1988. Characterization of a gene cluster for exopolysaccharide biosynthesis and virulence in *Erwinia stewartii*. *J. Bacteriol*. 170, 865–871. doi: 10.1128/jb.170.2.865-871.1988

Duong, DA, Jensen, RV, & Stevens, AM. 2018. Discovery of *Pantoea stewartii* ssp. *stewartii* genes important for survival in corn xylem through a Tn-Seq analysis. *Molecular Plant Pathology* 19(8):1929–1941. Advance online publication. https://doi.org/10.1111/mpp.12669

Duong DA, Stevens AM. 2017. Integrated downstream regulation by the quorum-sensing controlled transcription factors LrhA and RcsA impacts phenotypic outputs associated with virulence in the phytopathogen *Pantoea stewartii* subsp. *stewartii*. *PeerJ*. 5:e4145. doi:10.7717/peerj.4145

Grant SG, Jessee J, Bloom FR, Hanahan D. 1990. Differential plasmid rescue from transgenic mouse DNAs into *Escherichia coli* methylation-restriction mutants. *Proceedings of the National Academy of Sciences of the United States of America*. 87:4645–4649. doi: 10.1073/pnas.87.12.4645.

Kernell Burke A, Duong DA, Jensen RV, Stevens AM. 2015. Analyzing the transcriptomes of two quorum-sensing controlled transcription factors, RcsA and LrhA, important for *Pantoea stewartii* virulence. *PLoS ONE* 10:e0145358.

Kvitko, BH, Bruckbauer, S, Prucha, J, McMillan, I, Breland, EJ, Lehman, S, Mladinich, K, Choi, KH, Karkhoff-Schweizer, R, & Schweizer, HP. 2012. A simple method for construction of pir+ Enterobacterial hosts for maintenance of R6K replicon plasmids. *BMC Research notes* 5:157. https://doi.org/10.1186/1756-0500-5-157

Labes M, Puhler A, Simon R. 1990. A new family of RSF1010-derived expression and lac-fusion broad-host-range vectors for Gram-negative bacteria. *Gene* 89:37–46. doi: 10.1016/0378-1119(90)90203-4.

Monteiro, F, Genin, S, Dijk, I, & Valls, M. 2012. A luminescent reporter evidences active expression of *Ralstonia solanacearum* type III secretion system genes throughout plant infection. *Microbiology Society* 158:2107-2116. https://doi.org/10.1099/mic.0.058610-0

Stabb EV, Ruby EG. 2002. RP4-based plasmids for conjugation between *Escherichia coli* and members of the *Vibrionaceae*. *Methods in Enzymology*. 358:413–426. doi: 10.1016/S0076-6879(02)58106-4.

Stice, SP, Thao, KK, Khang, CH, Baltrus, DA, Dutta, B, Kvitko BH. 2020. *Pantoea ananatis* defeats Allium chemical defenses with a plasmid-borne virulence gene cluster. *bioRxiv* 2020.02.12.945675; doi: https://doi.org/10.1101/2020.02.12.945675
